# Active upper aerodigestive tract hemorrhage in patients with head and neck cancers: the “dot-in-sludge” sign

**DOI:** 10.1007/s10140-023-02118-9

**Published:** 2023-02-18

**Authors:** Abhishek Goswami, Ryan A. Fisicaro, Brian M. Howard, Milind Patel, Ashley H. Aiken, Kristen L. Baugnon, Xin Wu

**Affiliations:** 1 Department of Radiology, University of Chicago, Chicago, IL USA; 2 Department of Radiology and Biomedical Imaging, University of California San Francisco, San Francisco, CA USA; 3 Department of Neurosurgery, Emory University, Atlanta, GA USA; 4 Orlando Health, Orlando, FL USA; 5 Department of Radiology and Imaging Sciences, Emory University, Atlanta, GA USA

**Keywords:** Aerodigestive tract hemorrhage, Squamous cell cancer, Oropharyngeal, Oral cavity, Head and neck

## Abstract

Active extravasation into the upper aerodigestive tract is a dramatic and potentially life-threatening complication in patients with head and neck cancers. It prompts presentation to the emergency room and subsequent urgent imaging to identify the source of hemorrhage. Imaging of these patients may be complicated by treatment-altered anatomy, posing a challenge to the emergency radiologist who needs to rapidly identify the presence of active hemorrhage and the potential source vessel. This retrospective review summarizes the clinical and imaging findings of 6 oropharyngeal and oral cavity squamous cell cancer (SCC) patients with active upper aerodigestive tract hemorrhage. Most patients had advanced stage disease and prior radiation therapy. All CECT or CTA exams on presentation demonstrated the “dot-in-sludge” sign of active extravasation, as demonstrated by a “dot” of avidly enhancing extravasated contrast material layered against a background “sludge” of non-enhancing debris in the lumen of the upper aerodigestive tract. Common sources of hemorrhage included the lingual, facial, and superior thyroidal arteries. Familiarity with these findings will help radiologists increase their accuracy and confidence in interpreting these urgent, complex examinations.

## Introduction

Oropharyngeal and oral cavity squamous cell cancers (SCCs) affect over 54,000 new patients yearly in the USA [1]. Radiotherapy is a part of the treatment regimen for most oropharyngeal cancer patients, predisposing them to vasculopathies and hemorrhage [2]. Presenting as hemoptysis or hematemesis, upper aerodigestive tract hemorrhage is an airway-threatening emergency which prompts imaging with contrast-enhanced CT (CECT) or CT angiography (CTA) of the neck. Imaging is critical to identify a source of hemorrhage and potentially guide urgent endovascular or surgical treatment.

Given the inherent complexities of treatment-altered head and neck anatomy as well as the potential presence of tumor and radiation necrosis, interpretation of these exams may seem intimidating to any radiologist, especially when under the pressures of an emergent setting. The goal of this paper is to familiarize radiologists with characteristic findings of active aerodigestive tract hemorrhage and relevant external carotid artery (ECA) anatomy, so that one could accurately and rapidly detect the presence and source of hemorrhage.

## External carotid artery anatomy

**Fig. 1:**
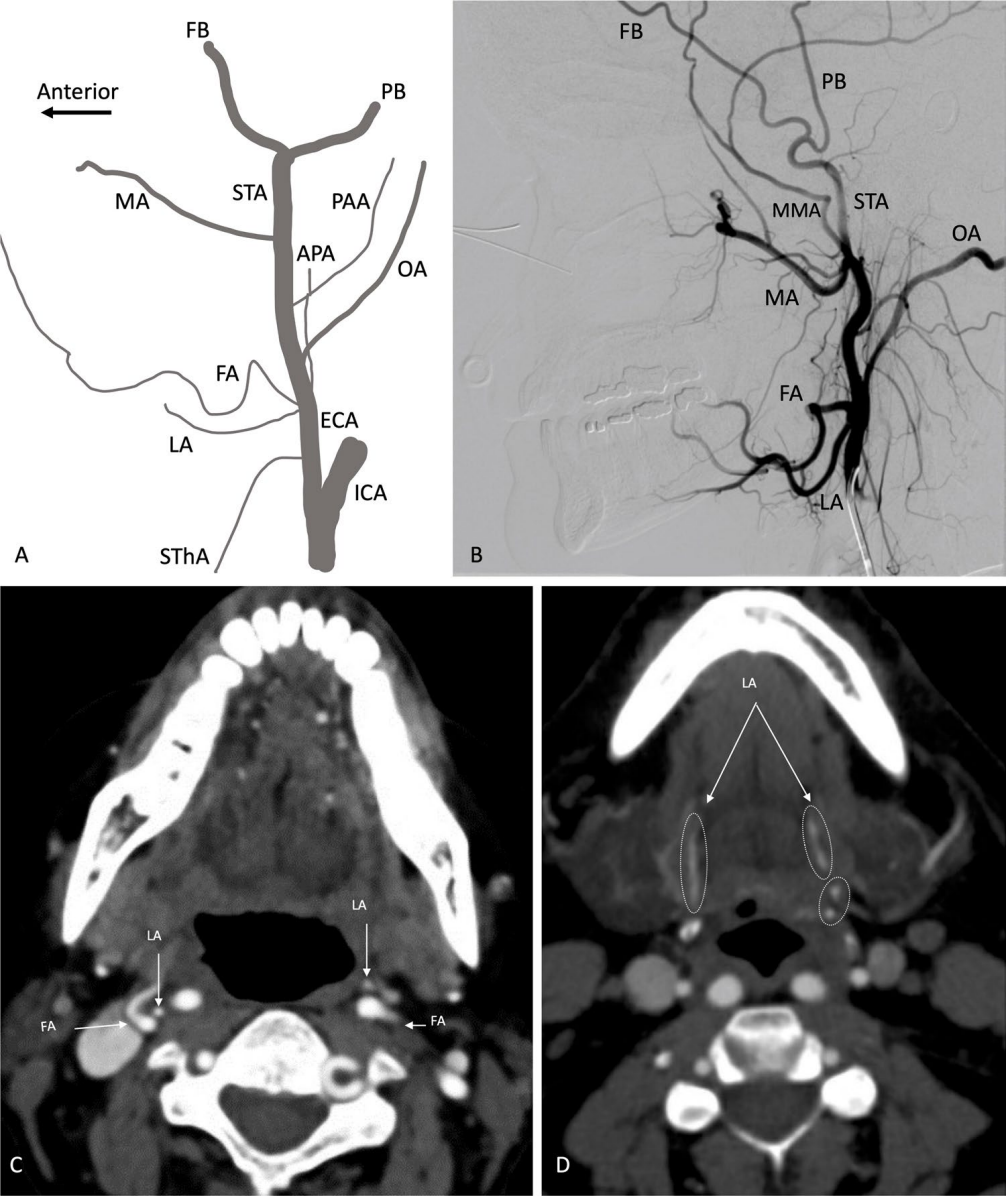
Major external carotid artery branches, as viewed in a lateral projection schematic diagram (**A**) and lateral DSA image (**B**). In **B**, the catheter is located beyond the origin of the ICA and SThA, which are not opacified on this image. Axial CTA images (**C** and **D**) demonstrate the close origins of the LA and FA from the ECA. ICA, internal carotid artery. ECA, external carotid artery. SThA, superior thyroid artery. LA, lingual artery. FA, facial artery. APA, ascending pharyngeal artery (projects medially). OA, occipital artery. PAA, posterior auricular artery. MA, maxillary artery. MMA, middle meningeal artery. STA, superficial temporal artery. FB, frontal branch. PB, parietal branch

Knowledge of ECA branch anatomy is important for patients with aerodigestive tract hemorrhage to detect the source of hemorrhage and to improve communication with surgeons and interventionalists (Fig. 1). The first branch of the ECA is the superior thyroid artery, projecting anterior with an inferior course. The second branch, the ascending pharyngeal artery, projects medially with a superior course. The next two anteriorly projecting branches, the lingual and facial arteries, have close origins which may be merged, with the lingual artery coursing along the lateral tongue while the facial artery has a tortuous course along the face. Posteriorly, the major branches include the occipital and relatively smaller posterior auricular arteries. The ECA then gives off the maxillary artery before terminating in the superficial temporal artery branches.

## Case series

An IRB-approved, HIPAA-compliant retrospective search of neck CECT and CTA reports from the past 15 years from a tertiary academic center was conducted. Patients with prior head and neck cancer suspected to have active hemorrhage in the upper aerodigestive tract on imaging were identified. Exclusion criteria included hemorrhage in the thoracic trachea, esophagus, or elsewhere in the neck. Patient imaging and medical records were reviewed to characterize imaging findings of active hemorrhage and key clinical features.

Six patients with head and neck SCC were identified (Table 1). Five patients had oropharyngeal SCCs treated at least partially by radiotherapy, while one had oral cavity SCC treated solely by surgery. Two patients had untreated recurrent oropharyngeal tumor at the time of acute hemorrhage, one patient was actively undergoing treatment for recurrence, and one patient had incompletely treated and persistent disease.

**Table 1:** Patient characteristics, history, presentation, hemorrhage localization, and outcome. *BOT* base of tongue,*CRT* chemoradiation therapy, *XRT*radiation therapy

Characteristics and features of six patients with active oropharyngeal hemorrhage								
Patient no	Age	Sex	Tumor staging and localization	Chief complaint	Therapy	Time from therapy to hemorrhage	CECT/CTA based source localization	Treatment/outcome
1	67	M	T3N1M0 Right BOT	Hemoptysis	Surgery and CRT	6 months	Right facial artery (CTA)	Transfusion, embolization of right lingual artery (facial artery occluded)
2	54	M	Unknown stage Left oropharynx	Hemoptysis	XRT alone	3 months	Left lingual artery (CTA)	Transfusion, embolization of left lingual/facial trunk
3	77	F	T3N1M0 Right tonsil with new right BOT recurrence	Hemoptysis	Surgery and CRT	28 years	Right facial or lingual artery (CECT)	Transfusion, embolization of right superior thyroid artery (facial and lingual arteries occluded)
4	64	F	T2aN3b Right retromolar trigone	Hematemesis	Surgery alone	1 week	Flap anastomosis (CECT)	Transfusion, self-resolved
5	67	M	rT4aN2c Right BOT after prior laryngeal SCC	Fever and epistaxis	Surgery and CRT	Ongoing CRT	Recurrent tumor bed (CECT)	Transfusion, self-resolved
6	71	M	T3N0 Right BOT with new left BOT recurrence	Hematemesis	Surgery and CRT	20 months	Recurrent tumor bed (CECT), left lingual artery (CTA)	Self-resolved, followed by oropharyngectomy and laryngectomy

All six patients were hospitalized, and five required transfusions. Active extravasation was visualized on imaging at the site of either the primary tumor or adjacent radiation field. Three patients required embolization, while three self-resolved after packing. The facial or lingual arteries were primary sources of suspected active extravasation.

### Case 1

**Fig. 2:**
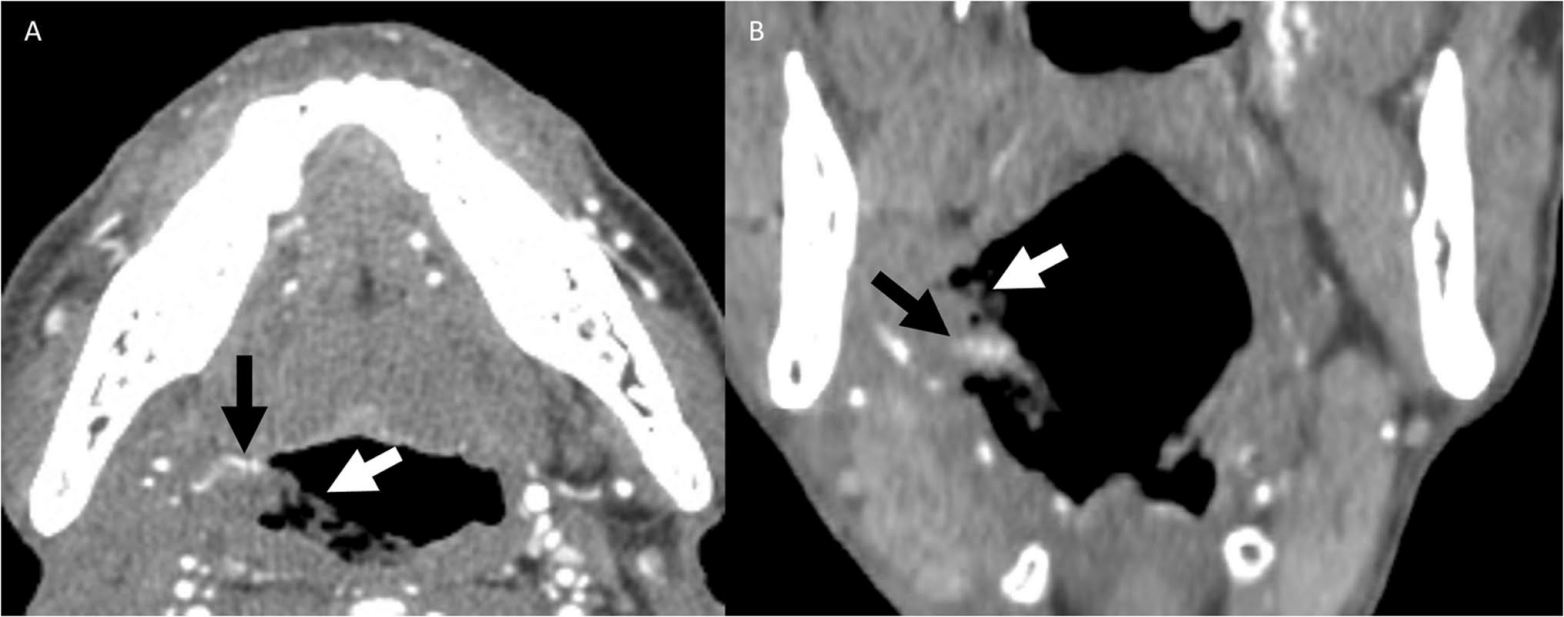
Patient 1. Axial (**A**) and coronal (**B**) postcontrast CTA images of the neck at the level of the base of tongue demonstrate linear high-density contrast material (black arrow) surrounded by low-density foamy debris (white arrow) in the right oropharyngeal lumen, compatible with active hemorrhage

**Fig. 3:**
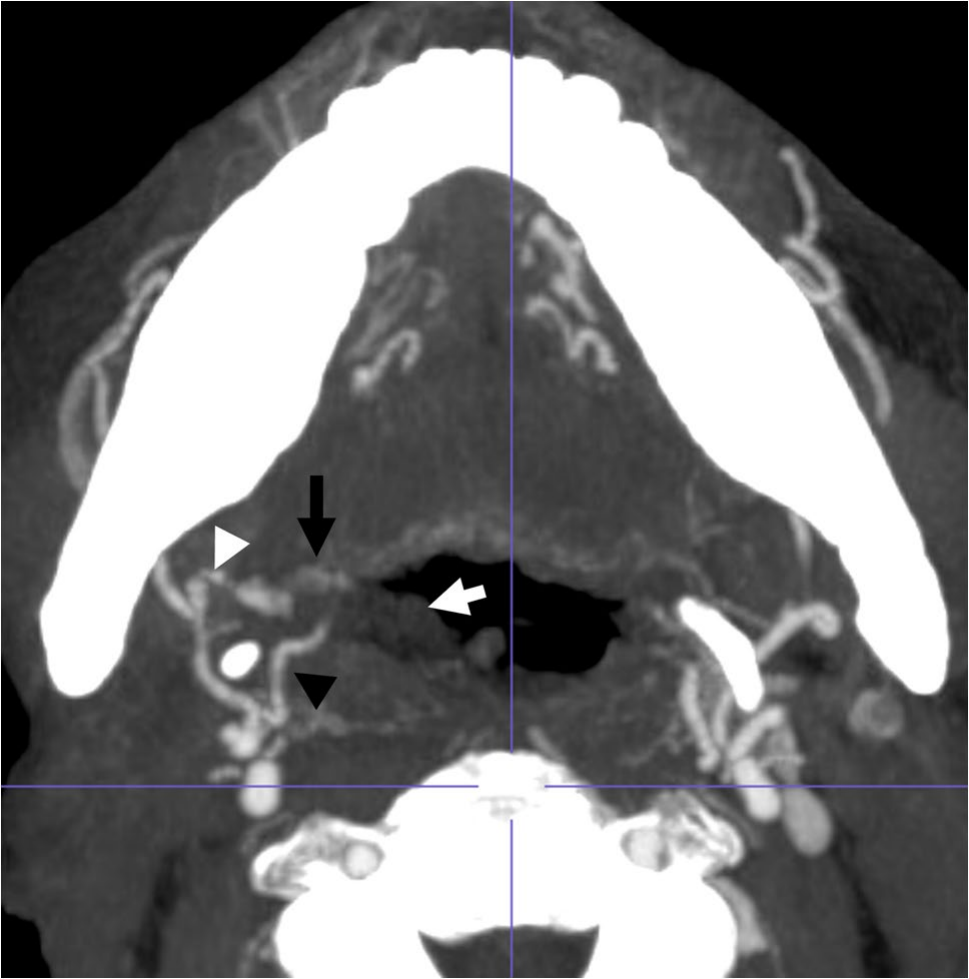
Patient 1. Axial postcontrast CTA MIP image of the neck at the level of the base of tongue demonstrates linear high-density contrast material in the right oropharyngeal lumen (black arrow) surrounded by low-density foamy debris (white arrow), appearing contiguous with a right facial artery branch (possibly ascending palatine or tonsillar) demonstrating irregular caliber changes (white arrowhead). The right lingual artery also appears to course into the intraluminal debris (black arrowhead)

**Fig. 4:**
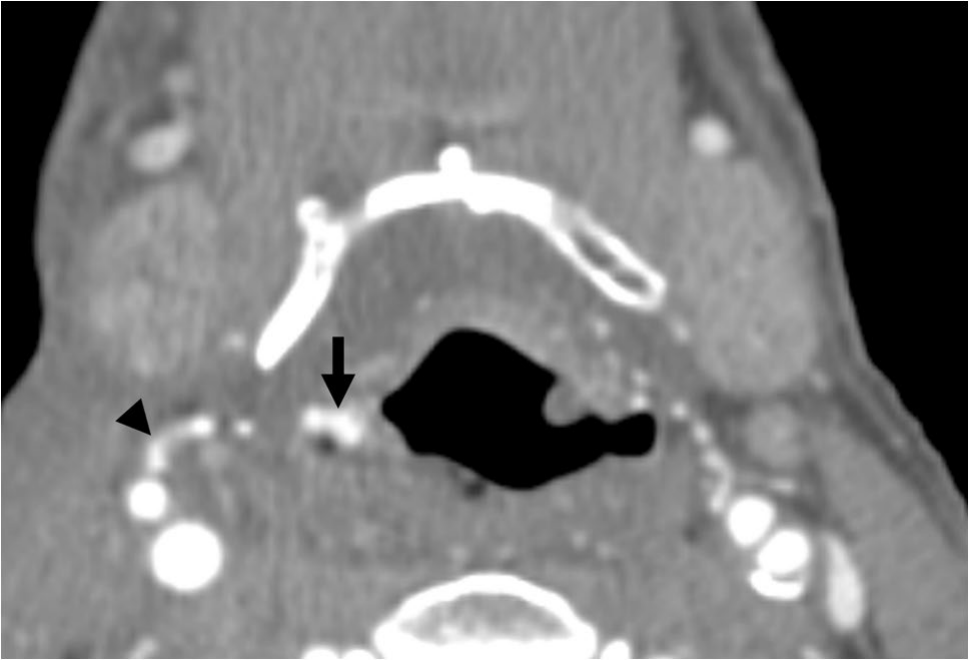
Patient 1. Axial postcontrast CTA image of the neck at the level of the pyriform sinuses demonstrates linear contrast material in the right pyriform sinus (black arrow), which appears to extend laterally towards an irregular appearing infrahyoid branch of the superior thyroidal artery (black arrowhead)

**Fig. 5:**
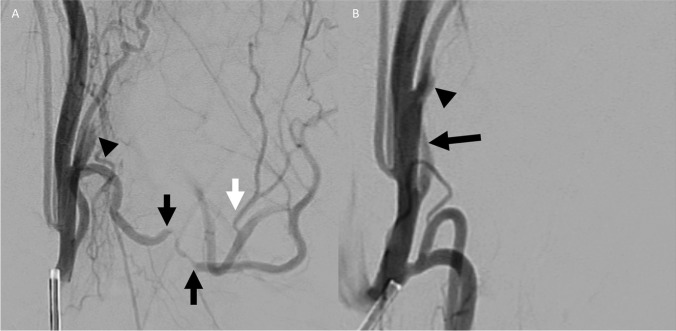
Patient 1. Lateral pre-embolization (**A**) DSA image of the neck with the catheter positioned in the proximal right ECA demonstrates occlusion of the proximal facial artery immediately after its origin (black arrowhead). The facial artery opacifies distally via anastomosis with the mylohyoid branch of the inferior alveolar artery (white arrow). The lingual artery shows evidence of significant radiation angiopathy (black arrows) and was the putative source of oropharyngeal bleeding. Catheter positioning in the ECA precludes evaluation of the superior thyroid artery branch. Lateral post-embolization (**B**) DSA image of the same view demonstrates persistent lack of opacification of the proximal facial artery (black arrowhead) and new occlusion of the proximal lingual artery following gel-foam embolization (black arrow). Catheter positioning in the ECA now allows for visualization of the superior thyroid artery branch (dotted black arrow)

A 67-year-old male with a history of T3N1 right base of tongue (BOT) oropharyngeal SCC presented with hemoptysis 6 months after completion of treatment. CTA demonstrated hypoattenuating intraluminal debris in the right oropharynx with a focus of contrast enhancement, compatible with active hemorrhage, contiguous with an irregular right facial artery branch (Figs. 2 and 3). A second pseudoaneurysm was present in the right piriform sinus, associated with an irregular infrahyoid branch of the superior thyroidal artery (Fig. 4). Patient required four units of PRBCs prior to DSA, which demonstrated nonopacification of the proximal right facial artery, presumably related to oropharyngeal packing, and an irregular right lingual artery, which was embolized with gel foam (Fig. 5).

### Case 2

**Fig. 6:**
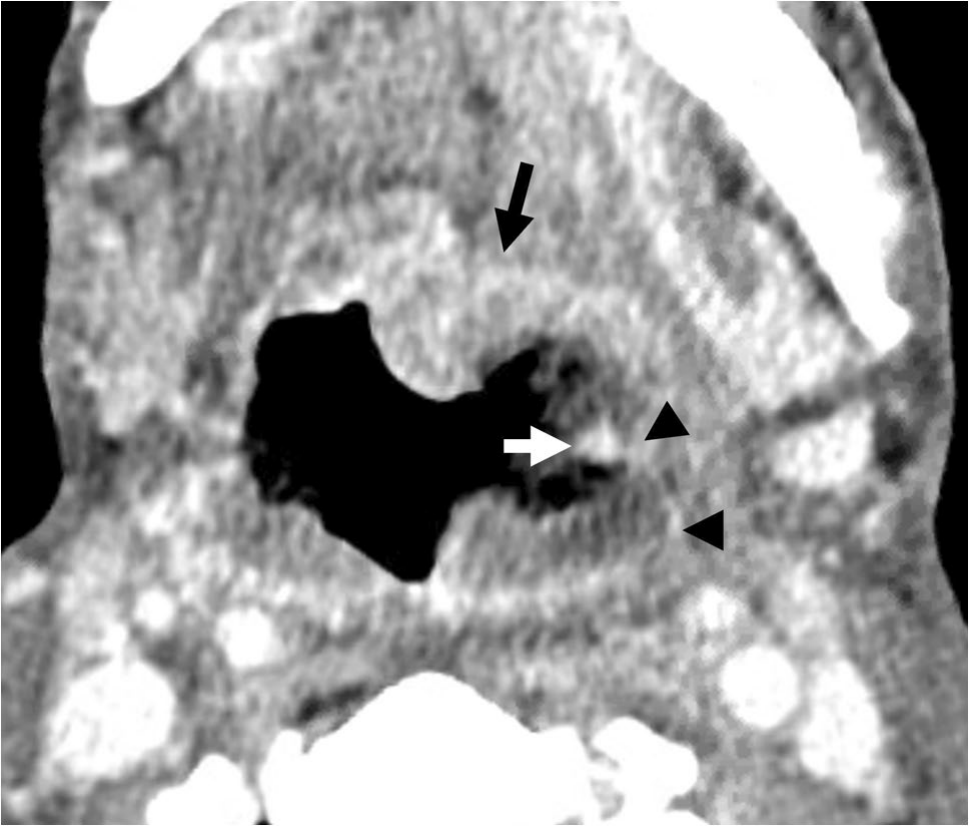
Patient 2. Axial CECT image at the level of the tongue base demonstrates a dot of hyperenhancement (white arrow) against a background of foamy nonenhancing luminal debris in the left oropharynx, compatible with active hemorrhage. Linear hyperenhancement (black arrowheads) extending from the focus of active hemorrhage corresponds to the distal left lingual artery. Ulcerated enhancing mass is present at the base of tongue (black arrow)

**Fig. 7:**
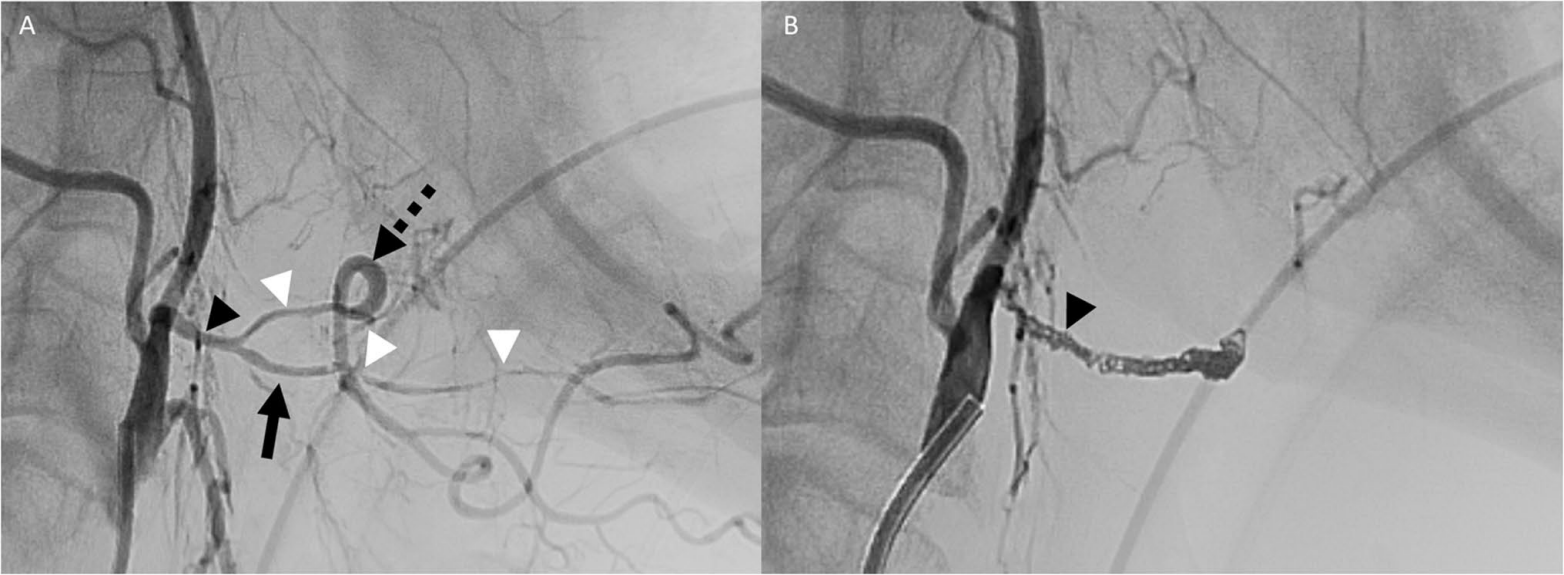
Patient 2. Lateral pre-embolization DSA image (**A**) of the neck with the catheter positioned in the proximal left ECA demonstrates a common trunk (black arrowhead) between the facial (dotted black arrow) and lingual (black arrow) branches. Irregular narrowing of the proximal facial artery and various portions of the lingual artery are also present (white arrowheads). Lateral post-embolization DSA image (**B**) of the neck with the catheter positioned in the proximal left ECA demonstrates embolization of the facial and lingual arteries, which was completed with polyvinyl alcohol particles followed by coils from the lingual artery into the common facial-lingual trunk (black arrowhead)

A 54-year-old male with a history of recurrent left oropharyngeal SCC presented with hemoptysis after incomplete radiation therapy 3 months prior. CECT showed a necrotic left oropharyngeal mass with signs of active hemorrhage adjacent to an irregular left lingual artery (Fig. 6). Patient required four units of PRBCs before undergoing DSA, which demonstrated a common trunk of the left facial and lingual arteries and irregularity of these arteries near the area of extravasation (Fig. 7). Coil embolization of the common trunk was performed along with polyvinyl alcohol embolization of the left lingual artery, with cessation of hemorrhage (Fig. 7).

### Case 3

**Fig. 8:**
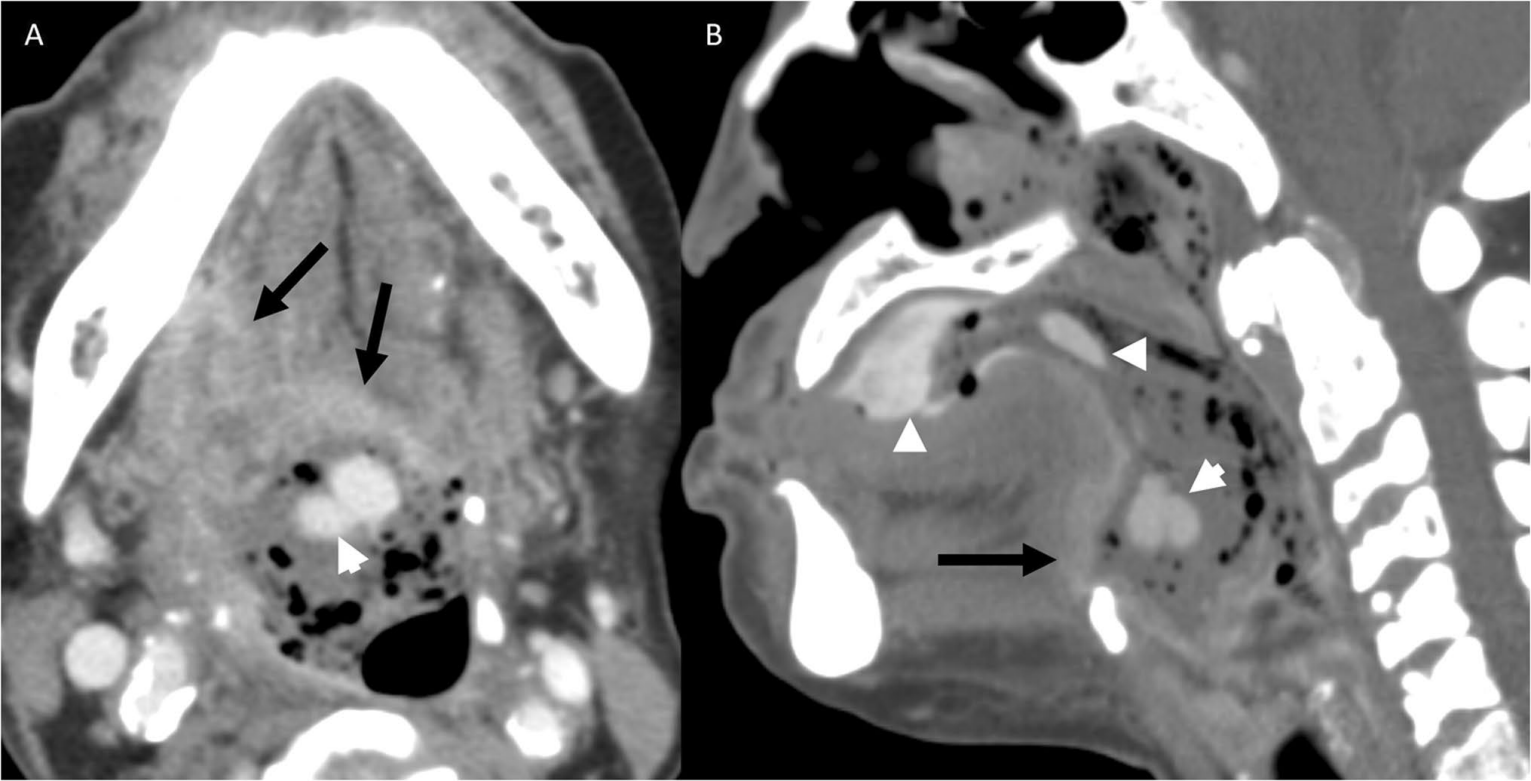
Patient 3. Postcontrast CECT images of the neck in axial plane at the level of the base of tongue (**A**) and midline sagittal plane (**B**) demonstrate multiple foci of contrast material (white arrowheads) surrounded by foamy debris throughout the oral cavity and oropharyngeal lumen, compatible with active hemorrhage. Additionally, a heterogeneously enhancing ulcerating mass is seen at the tongue base, extending endophytically into the right floor of mouth and glossotonsillar sulcus, compatible with tumor recurrence (black arrows)

**Fig. 9:**
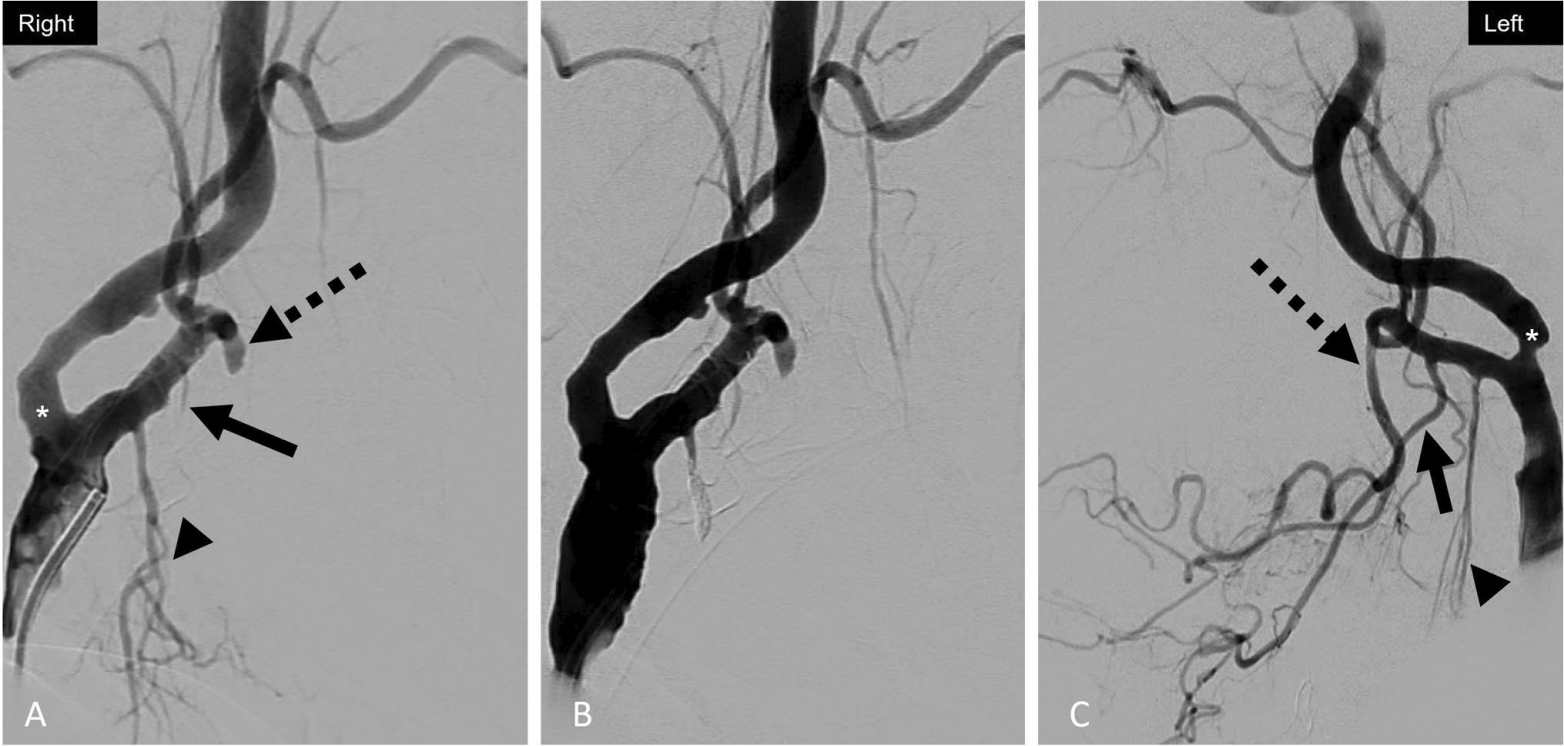
Patient 3. Lateral pre-embolization DSA images of the neck with the catheter positioned in the common carotid artery on the right (**A**) demonstrates occlusion of the right facial (dotted black arrow) and lingual (black arrow) arteries, presumably related to prior packing. Irregular narrowing and tortuosity of the right superior thyroid artery branches are noted (black arrowhead). The terminal branches of the superior thyroid artery ended in abnormal tumor blush believed to be the source of hemorrhage. Given concern for reflux of particles into the ICA, the superior thyroid artery was sacrificed (**B**) with coils (white arrow). The left common carotid angiogram (**C**) is provided for comparison with annotations of the facial (dotted black arrow), lingual (black arrow), and superior thyroid (black arrowhead) arteries. Irregularities of the bilateral carotid bifurcations and proximal ICA lumens (white asterisks) are presumably related to underlying atherosclerotic disease

A 77-year-old female with a history of T3N1 right tonsillar SCC treated with surgery and CRT 28 years prior presented with hemoptysis. CECT neck demonstrated a recurrent right oropharyngeal mass, active hemorrhage in the oral cavity and oropharynx, and irregularity of the right ECA and lingual arteries (Fig. 8). The patient required one unit of PRBCs and packing prior to DSA. DSA showed nonopacification of the right facial and lingual arteries, presumably related to packing, and irregularity of the right superior thyroid artery, successfully treated with coil embolization (Fig. 9).

### Case 4

**Fig. 10:**
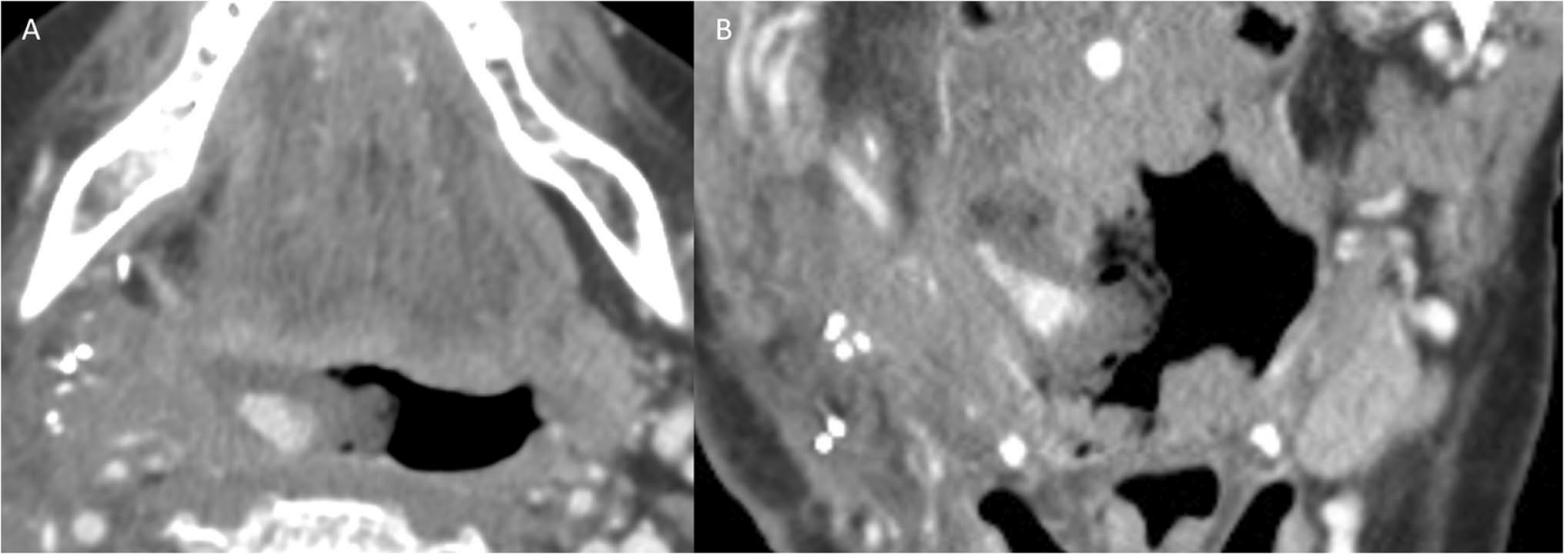
Patient 4. Postcontrast CECT images of the neck in axial plane at the level of the base of tongue (**A**) and in coronal plane at the level of the oropharynx (**B**) demonstrate a focus of contrast material (black arrow) surrounded by foamy debris in the right oropharyngeal lumen (white arrow), compatible with active hemorrhage. The area of hemorrhage is closely located to the inferior margin of the radial forearm free flap anastomosis, as indicated by soft tissue thickening and surgical clips (black arrowheads)

A 64-year-old female with a history of T2aN3b oral cavity SCC of the right retromolar trigone (RMT) presented with hematemesis 1 week after undergoing surgical resection and reconstruction. CECT demonstrated active hemorrhage in the right oropharyngeal lumen, near the presumed anastomotic site at the posteroinferior flap margin (Fig. 10). While there was multifocal irregularity of the right ECA, a specific source of hemorrhage could not be localized. The patient required two units of PRBCs and the hemorrhage self-resolved.

### Case 5

**Fig. 11:**
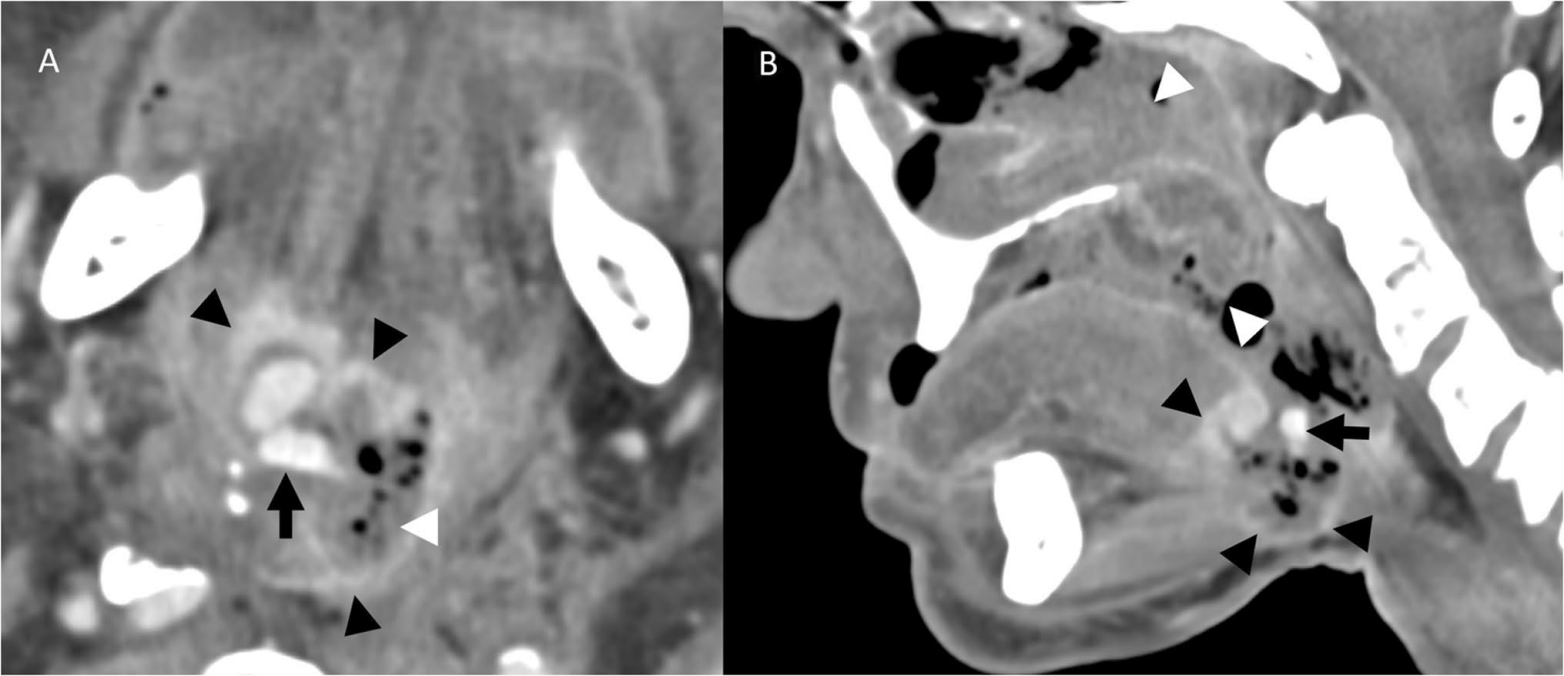
Patient 5. Postcontrast CECT images of the neck in axial plane at the level of the base of tongue (**A**) and in mid sagittal plane (**B**) show an enhancing ulcer at the BOT extending towards the skin surface (black arrowheads), with an area of right oropharyngeal intraluminal enhancement immediately adjacent to the enhancing BOT soft tissue (black arrow) compatible with acute hemorrhage. Foamy debris fills the oropharynx, oral cavity, and nasopharynx (white arrows)

A 67-year-old male with a remote history of laryngeal SCC treated with total laryngectomy and CRT developed a right BOT anastomotic site recurrence 15 months prior and presented with fever and epistaxis while on CRT. CECT showed a recurrent tumor at the BOT with a pharyngocutaneous fistula (Fig. 11). An area of active hemorrhage was presented immediately adjacent to the BOT tumor bed. Non-enhancing debris extended to the nasopharynx, compatible with reflux of blood products from the oropharynx. The patient required 1 unit of PRBCs, and the hemorrhage self-resolved.

### Case 6

**Fig. 12:**
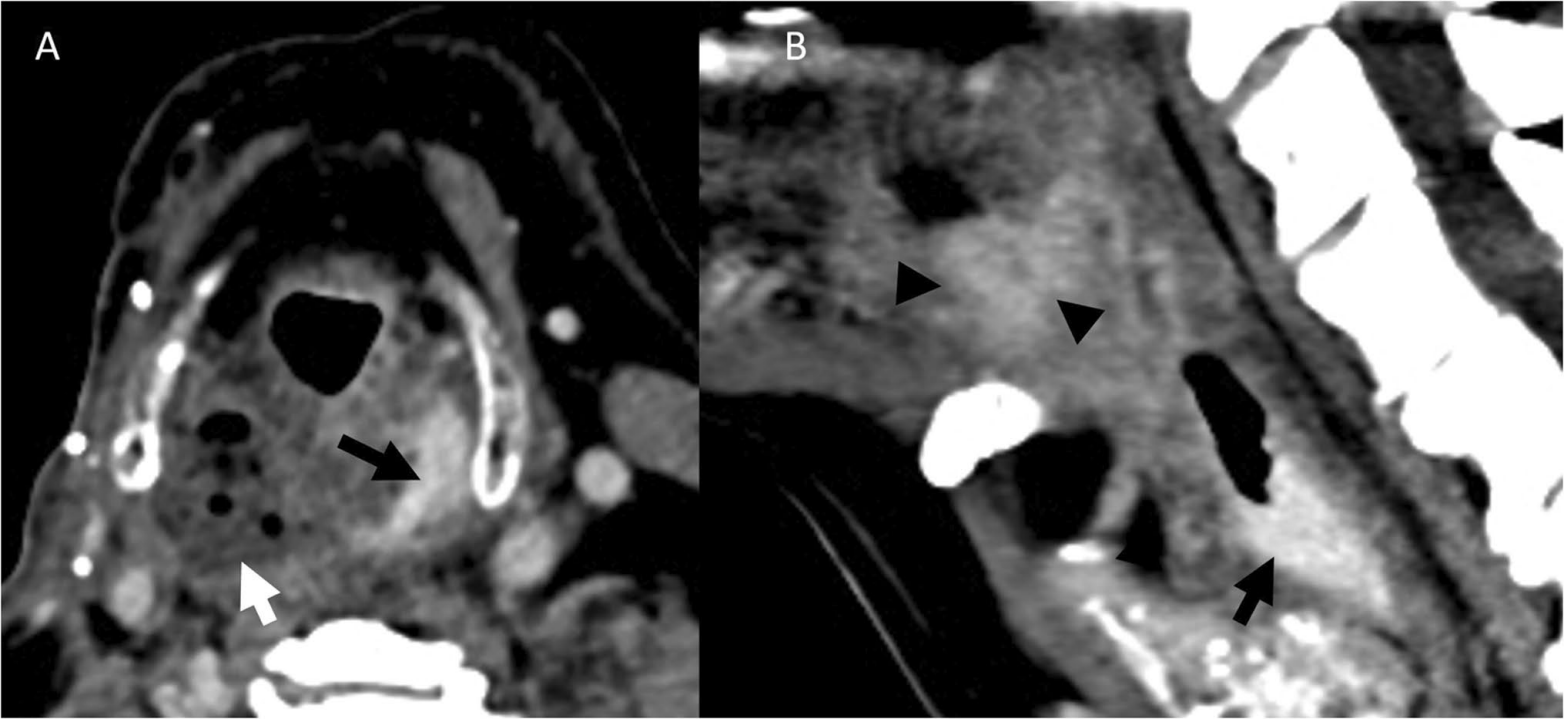
Patient 6. Postcontrast CECT images of the neck in axial plane at the level of the aryepiglottic folds (**A**) and in sagittal plane at the level of the left pyriform sinus (**B**) show left pyriform intraluminal contrast (black arrows) with foamy debris in the contralateral pyriform sinus (white arrow). Enhancing soft tissue in the left BOT is compatible with recurrent tumor (black arrowheads)

**Fig. 13:**
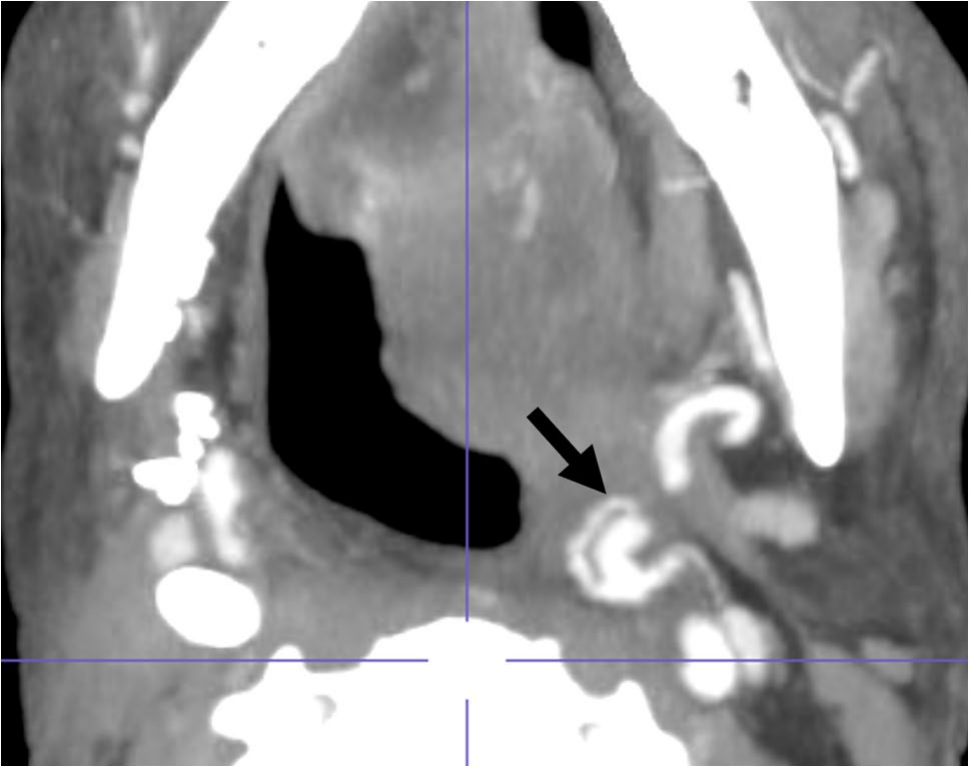
Patient 6. Axial CTA MIP image of the neck at the level of the BOT obtained 10 days following acute presentation demonstrates an irregular appearing left lingual artery (black arrow) extending into the left BOT recurrent tumor bed, likely the source of hemorrhage

A 71-year-old male with a history of T4N2B right oropharyngeal SCC, originally treated 9 years prior with CRT, developed recurrent disease in the right BOT which was treated with surgery one year prior. The patient presented with hematemesis, and CECT showed recurrent tumor in the BOT and contralateral left hypopharynx, with active contrast extravasation in the left piriform sinus which subsequently self-resolved (Fig. 12). Repeat CTA 10 days later showed a pseudoaneurysm of the left lingual artery within the tumor bed (Fig. 13).

## Discussion

Active extravasation into the upper aerodigestive tract is a potentially life-threatening complication in patients with treated or recurrent head and neck cancers. Existing literature is limited to case reports and focuses on its treatment and management rather than imaging diagnosis [3–7]. In this series, we demonstrate that the “dot-in-sludge” sign indicates active extravasation within the upper aerodigestive tract, as demonstrated by a “dot” of extravasated contrast material against a background “sludge” of non-enhancing foamy intraluminal secretions and debris. This finding should prompt radiologists to search for a potential arterial source vessel within the soft tissues immediately adjacent to the “dot” of extravasation.

Although active extravasation can be identified on both CECT and CTA, CTA is the modality for detecting its source [8, 9]. Cases with identifiable sources of bleeding are prime candidates for endovascular embolization, whereas those without a source are generally treated conservatively, as indiscriminate embolization is potentially futile and may undermine treatment options in the event of future hemorrhage. The mucosal surfaces of the oral cavity and oropharynx are supplied by branches of the ECA. In this series, CTA identified specific ECA branches as suspected sources of hemorrhage, while CECT only identified vascular irregularities at the level of the ECA. Signs of vascular damage included abrupt vessel caliber changes and beading, pseudoaneurysms, and vascular occlusion. Irregularities were predominantly noted in the lingual and facial branches, which supply the base of tongue.

In the setting of head and neck cancer, vascular complications occur most commonly due to radiation vasculopathy or invasion by hypervascular, friable tumor [10–12]. In this series, five of the six hemorrhages occurred within 20 months of radiation. Among patients with prior radiation treatment, increased risk of hemorrhage is associated with advanced T category, persistent disease, and radiation necrosis [13]. Four of six patients in this study had T3 or T4 tumors, and all patients had clinical histories consistent with progressive or persistent disease.

Accurate rapid detection of upper aerodigestive tract extravasation is critical in both preventing further deterioration of an unstable patient and localizing the source vessel as a potential treatment target. This is especially important in advanced stage oropharyngeal and oral cavity SCC patients who are at higher risk of having this complication. The “dot-in-sludge” sign, indicating active contrast extravasation against a background of non-enhancing blood products and debris, is an emergent imaging finding that should alert radiologists of a nearby source vessel. Familiarity with ECA branch anatomy is crucial to help detect the source of hemorrhage and guide further treatment decision-making.
